# *Schizochytrium* Supplementation in Compound Feed: Effects on Growth, Metamorphosis, Intermediate Metabolism, and Intestinal Health of Bullfrogs (*Lithobates catesbeianus*)

**DOI:** 10.3390/antiox14101208

**Published:** 2025-10-05

**Authors:** Hao Ding, Yinglin He, Yujian Song, Jingjing Liang, Woxing Li, Chao Xu, Huirong Yang

**Affiliations:** 1 College of Marine Sciences, South China Agricultural University, Guangzhou 510642, China; haoding@stu.scau.edu.cn (H.D.); yinglinhe@stu.scau.edu.cn (Y.H.);; 2 Zhongshan Innovation Center, South China Agricultural University, Zhongshan 528400, China

**Keywords:** *Schizochytrium* meal, metamorphosis, bullfrog nutrition, intestinal health

## Abstract

*Schizochytrium* is often added to feed to enhance the growth and health of farmed animals, yet research on its effects on amphibians remains relatively scarce. Here, this study investigated the effects of dietary *Schizochytrium* meal on growth, metamorphosis, intermediate metabolism, and intestinal health of bullfrogs. Six compound feeds (S0–S5) containing different gradients of *Schizochytrium* meal (0.00, 2.00, 5.00, 10.00, 15.00, and 20.00 g/kg diets) were formulated. After 90 days, the S4 group (15.00 g/kg) exhibited significantly superior growth performance, with the weight gain rate (WGR) increasing by up to 23.78% compared to the control (S0). Metamorphosis rate (MR) peaked at 23.33% in the S4 group. The enzyme activities of digestion (amylase (AMS), lipase (LPS), protease), brush border membrane (Na^+^, K^+^-ATPase, alkaline phosphatase (AKP), γ-glutamyl transferase (γ-GT), creatine kinase (CK), and antioxidation (superoxide dismutase (SOD), catalase (CAT)), as well as microvilli length and mucosal epithelial cell height in the intestine were the highest in the S4 group. Intestinal microbial diversity (Ace index) significantly increased by 41.28% in the S4 group, which also promoted beneficial bacteria. Key genes related to the GH-IGF-1 axis, metabolism, and intestinal barrier function were significantly upregulated with increasing *Schizochytrium* levels up to 15.00 g/kg, whereas pro-inflammatory genes showed an opposite trend. Overall, dietary supplementation with *Schizochytrium* meal at 15.00 g/kg promotes growth, metamorphosis, and intestinal health in bullfrog tadpoles by modulating the GH-IGF-1 axis, enhancing digestion and absorption, and improving intestinal integrity. Optimal *Schizochytrium* meal levels were identified as 13.27 g/kg.

## 1. Introduction

With the increasing demand of consumers for healthy white meat products, bullfrog (*Lithobates catesbeianus*) farming has great market potential. A critical stage in bullfrog production is tadpole rearing, as it directly influences the health and growth performance of subsequent juvenile frogs [1]. This phase is characterized by metamorphosis, a complex developmental process involving the transformation of aquatic tadpoles into amphibious frogs, marked by fundamental remodeling of tissues and organs such as the development of limbs and lungs [2,3,4]. Therefore, the efficiency of this metamorphic process is a key determinant of overall breeding success, highlighting the need for nutritional strategies to support tadpole development.

Food nutrition is a key factor to guarantee high breeding efficiency of farmed animals. The marine microalgae *Schizochytrium* has emerged as a promising aquafeed ingredient, renowned for its high content of polyunsaturated fatty acids (PUFAs), particularly docosahexaenoic acid (DHA) [5]. This microalga is commonly used as a live food source for the larval and juvenile stages of aquatic animals due to its high nutritional value and suitable cell size [6]. Its nutritional value has been well-documented in various fish species, where dietary supplementation has been shown to improve growth performance, feed utilization, stress tolerance, and disease resistance [7,8]. Specifically, dietary supplementation with *Schizochytrium* significantly reduced the feed conversion ratio to 1.20% in golden pompano (*Trachinotus ovatus*) and zebrafish (*Danio rerio*), while also improving non-specific immunity and increasing the abundance of beneficial intestinal bacteria [9,10]. In Nile tilapia (*Oreochromis niloticus*), *Schizochytrium* completely replaced fish oil in feed, enhancing weight gain, feed efficiency, protein efficiency, and optimizing the DHA:EPA ratio in filets, demonstrating that *Schizochytrium* modulates growth performance and fatty acid metabolism in aquatic species [11]. Furthermore, *Schizochytrium* upregulates the expression of antioxidant and immune-related genes in rainbow trout (*Oncorhynchus mykiss*) [12]. However, the existing body of evidence is predominantly derived from fish, and direct evidence for its application in amphibians is currently lacking.

The role of DHA in amphibian development, particularly during the critical window of metamorphosis, warrants greater attention. As in other vertebrates, DHA is recognized for its crucial role in neural development and visual function in amphibians [13,14,15]. Adequate lipid nutrition, including a balanced provision of essential fatty acids, is vital to support the extensive tissue remodeling and high energy demands characteristic of metamorphosis [16], but the specific requirements and optimal levels of fatty acids for amphibians are not well-defined. Most existing inferences are drawn from fish or general vertebrate models [17].

Beyond systemic growth, intestinal health is paramount for nutrient acquisition and overall development. A well-functioning intestine, supported by a balanced interaction between the gut microbiota, epithelial barrier, and immune system, is essential for efficient digestion and absorption [18,19,20,21]. In addition, the enzyme activity in the intestine is the reliable indicator for evaluating intestinal absorption and digestion capacity, due to the fact that these enzymes affect the ability of animals to obtain and utilize nutrients in food [22,23]. While the positive impacts of *Schizochytrium* on intestinal health have been observed in fish, its influence on the gut function of amphibian tadpoles, particularly during the metabolically demanding period of metamorphosis, remains uninvestigated.

To address these research gaps, the present study was designed to systematically evaluate the effects of dietary *Schizochytrium* meal supplementation on growth, metamorphosis, intermediary metabolism, and intestinal health in bullfrog tadpoles over a 90-day feeding trial. We hypothesized that dietary *Schizochytrium* meals would promote tadpole growth and metamorphosis by enhancing digestive function, modulating key metabolic pathways, and improving intestinal health. The findings are expected to provide novel insights into the application of *Schizochytrium* in amphibian aquaculture and contribute to developing effective nutritional strategies for improving bullfrog breeding efficiency.

## 2. Materials and Methods

### 2.1. Ethics Statement

The Animal Care and Use Committee in South China Agricultural University have approved the present study (Permit No.: 2017D006).

### 2.2. Diets, Bullfrog, and Sampling

*Schizochytrium* meal was obtained from Guanxing Agricultural Technology Co., Ltd. (Guangzhou, China). Graded doses of *Schizochytrium* meal (0, 2.00, 5.00, 10.00, 15.00, and 20.00 g/kg) were added to the basal diet (Table 1). *Schizochytrium* meal doses were selected according to the research on *Ictalurus punctatus* and *Lateolabrax maculatus* [24,25]. The diets were made by a granulation machine (Beijing Modern Yanggong Machinery Sand Development Co., Ltd., Beijing, China).

Bullfrog tadpoles were obtained from Guanxing Agricultural Technology Co., Ltd. (Guangzhou, China). A total of 720 tadpoles (0.04 ± 0.00 g) were dispersed into 18 indoor tanks (40 L) with equal individuals (40/tank). Temperature, dissolved oxygen, pH, and ammonia–nitrogen were monitored daily and maintained within optimal ranges for bullfrog tadpole farming [26]. Six compound feeds (S0–S5) were fed to tadpoles four times a day (08:30, 12:00, 17:30, and 22:00). After 90 days, total number, weight, and body length of bullfrogs in each tank were recorded for the analysis of weight gain rate, specific growth rate, and condition factor. Number of tadpoles with hind limb buds was recorded for the analysis of post-premetamorphosis rate (PPR) and metamorphosis rate (MMR). A total of 10 tadpoles from each tank were sampled for the analysis of proximate composition. Four tadpoles per tank were anesthetized via MS-222 at 100 mg/L, and brain, liver, and intestine samples were obtained for PCR and biochemical analysis. In addition, four tadpoles from each tank were anesthetized to collect intestine samples for the analysis of intestinal microbiota and hematoxylin–eosin (HE) staining.

### 2.3. Analytic Procedures

#### 2.3.1. Analysis of Proximate Composition and Intestinal Histomorphometry

The proximate composition of compound feeds and tadpoles were determined by AOAC protocols [15]. Intestinal HE staining was carried out via the methods of Xu et al. [16]. In short, middle intestine samples were sectioned using a slicing machine (RD-2230, Shenyang Roundfin Technology, Co., Ltd., Shenyang, China) and were then stained by hematoxylin and eosin (HE) staining kit (NO. S20202-2×100, Shanghai Shangbao Biotechnology Co., Ltd., Shanghai, China). The images were obtained via a light-polarizing microscope (BX53P, Olympus, Tokyo, Japan).

#### 2.3.2. Analysis of Intestinal Biochemical Indicators and Microbiota

Intestinal amylase (AMS, C016-1-1), lipase (LPS, A054-2-1), protease (A080-1-1), Na^+^, K^+^-ATPase (A070-2), alkaline phosphatase (AKP, A059-2), γ-glutamyl transferase (γ-GT, C017-2-1), creatine kinase (CK, A032-1-1), superoxide dismutase (SOD, A001-3), catalase (CAT, A007-1-1), total antioxidant capacity (T-AOC, A015-3-1), and malondialdehyde (MDA, A003-1) were detected using commercial kits (Jiancheng Biotech, Co., Ltd., Nanjing, China).

Bacterial DNA was extracted from intestine via the QiAamp DNA stool Mini Kit (Qiagen, Dusseldorf, NRW, Germany). DNA quality and concentration were assessed with a NanoDrop2000 spectrophotometer (Thermo Fisher Scientific, Waltham, MA, USA). The V3-V4 regions of the bacterial 16S rRNA gene were amplified using primers 338F (5′-ACTCCTACGGGAGGCAGCAG-3′) and 806R (5′-GGACTACHVGGGTWTCTAAT-3′). PCR was performed with denaturation at 94 °C for 2 min, followed by 30 cycles (98 °C for 10 s, 55 °C for 30 s, 68 °C for 30 s) and a final extension at 68 °C for 5 min. Purified amplicons were sequenced using a 2 × 300 bp paired-end format on the Illumina MiSeq platform (Illumina, San Diego, CA, USA). The sequence data have been deposited at NCBI GenBank database under accession number PRJNA1335207.

The qualified raw sequence data was split based on barcodes and the primer and adapter sequences were removed using the cutadapt plugin in QIIME2 (version 2024). Paired-end reads were merged based on their overlap relationship to obtain optimized data after quality control. Chimera sequences were removed and clustered into amplicon sequence variants (ASVs) using DADA2 (version 2024). A total of 1,286,881 raw sequences were obtained from bacterial samples, and subsequent processing of high-quality reads identified 2491 bacterial ASVs. Species annotation was performed using the classifier-sklearn plugin with the Silva-138 reference database (https://www.arb-silva.de/, accessed on 15 August 2025). Community structure at both the phylum and genus levels was analyzed using Python software (version 2.7). Alpha diversity indices were calculated using QIIME2. Beta diversity was analyzed with principal coordinate analysis (PCoA) and non-metric multidimensional scaling (NMDS) based on Bray–Curtis dissimilarity using Vegan package (version 2.5-3), with significance tested through permutation multivariate analysis of variance (Adonis).

#### 2.3.3. Analysis of Polymerase Chain Reaction (PCR)

PCR analysis was also performed following the methods previously described by Xu et al. [16]. Briefly, total RNA was extracted from brain, liver, and intestine samples by Total RNA Isolation Kit (Vazyme Biotech Co., Ltd., Nanjing, China). The cDNA was synthesized using the HiScript IV 1st Strand cDNA Synthesis Kit (Vazyme, Nanjing, China) and T100 thermal cycler (Bio-rad, Hercules, CA, USA). RT-qPCR assays were carried out on a CFX Duet RT-PCR System (Bio-rad, Hercules, CA, USA) using Taq Pro Universal SYBR qPCR Master Mix (Vazyme, Nanjing, China). The primers of the target genes are presented in Table 2. *β-actin* was used as the internal reference gene, and the relative expression levels of the target gene were determined using the 2^−ΔΔCT^ method [27].

### 2.4. Statistical Analysis

All statistical analyses were performed through SPSS program (version 22.0). The normality of the data was confirmed using the Shapiro–Wilk test, and the homogeneity of variances was confirmed using Levene’s test. One-way ANOVA (SPSS program version 22.0) followed by Tukey’s multiple comparison test was used to identify differences among all experimental groups. Data are presented as mean ± standard error (mean ± S.E.M) of the mean, and *p* < 0.05 is significantly different.

## 3. Results

### 3.1. Growth Performance, Feed Utilization, and Metamorphosis Rate

**Figure 1:**
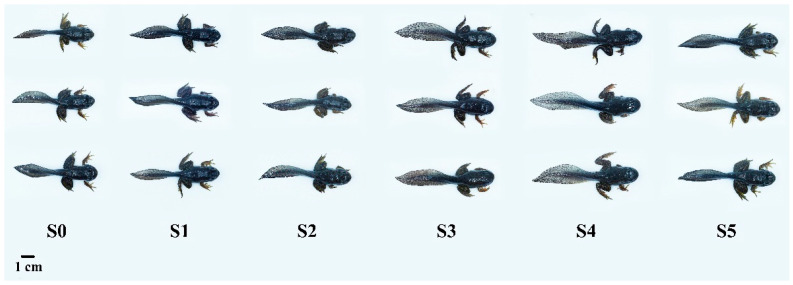
The photographs of bullfrog tadpoles after 90 days.

**Figure 2:**
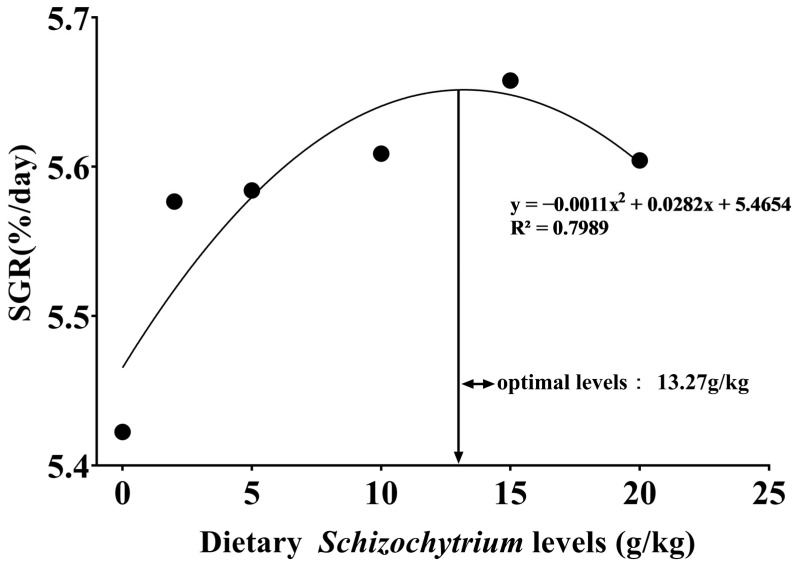
Optimal levels for bullfrog tadpoles based on specific growth rate (SGR) as determined by the broken line model.

The condition factor, whole-body moisture, and ash contents showed no significant differences (*p* > 0.05) among all groups (Table 3). Final weight, weight gain rate (WGR) (Figure 1), specific growth rate (SGR), feed intake (FI), PPR, and MR increased significantly (*p* < 0.05) as the *Schizochytrium* levels increased to 15.00 g/kg diet but decreased with further increasing *Schizochytrium* levels. Feed conversion ratio (FCR) declined obviously (*p* < 0.05) as *Schizochytrium* levels increased to 15.00 g/kg diet but increased with further increasing *Schizochytrium* levels. The highest contents of whole-body crude protein and lipids were observed in diets S5 and S3, respectively. Based on the quadratic regression analysis of SGR against *Schizochytrium* levels, optimal *Schizochytrium* levels for bullfrog tadpoles were estimated to be 13.27 g/kg diet (Figure 2).

### 3.2. Intestinal Biochemical Indicators and Histomorphometry

**Figure 3:**
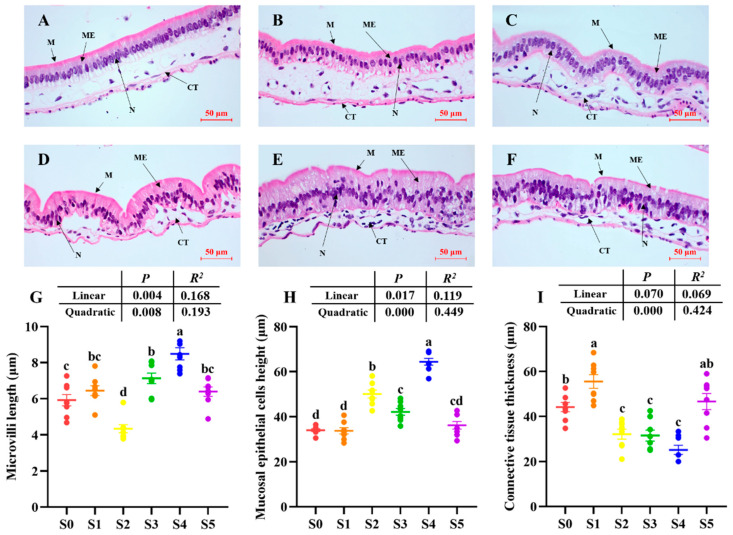
Longitudinal sections of the intestine of bullfrog tadpoles subjected to different treatments. (**A**) S0; (**B**) S1; (**C**) S2; (**D**) S3; (**E**) S4; (**F**) S5; (**G**) microvilli length; (**H**) mucosal epithelial cells height; (**I**) connective tissue thickness; CT, connective tissue; ME, mucosal epithelial cells; M, microvilli; N, cell nucleus. Scale bars = 50 μm. Values are mean ± S.E.M of three replications (n = 8). Values with the different superscript letters are significantly different among groups (*p* < 0.05).

Total antioxidant capacity showed no significant differences (*p* > 0.05) among all groups (Table 4). The activities of AMS, LPS, PES, Na^+^, K^+^-ATPase, AKP, γ-GT, CK, SOD, and CAT, as well as microvilli length and mucosal epithelial cell height in the intestine increased obviously (*p* < 0.05) as the *Schizochytrium* levels increased to 15.00 g/kg diet but decreased with further increasing *Schizochytrium* levels. Malondialdehyde contents and connective tissue thickness in the intestine declined obviously (*p* < 0.05) as *Schizochytrium* levels increased to 15.00 g/kg diet but increased with further increasing *Schizochytrium* levels (Figure 3).

### 3.3. Analyses of Intestinal Microbiota

**Figure 4:**
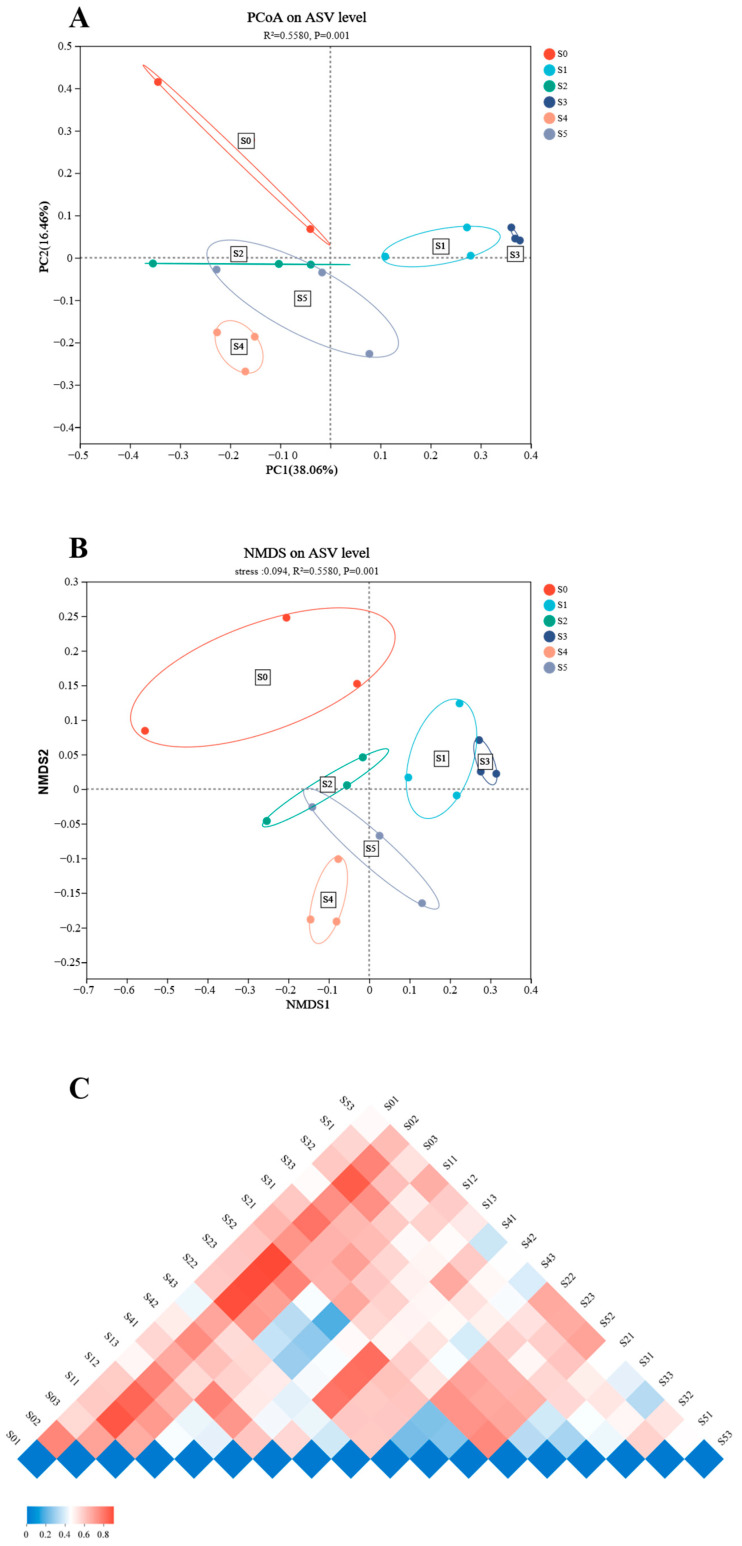
The beta diversity of intestinal microbiota of bullfrog tadpoles subjected to different treatments (Adonis, R^2^ = 0.5580, *p* = 0.001). (**A**), Principal co-ordinates analysis (PCoA). (**B**), Non-metric multidimensional scaling (NMDS). (**C**), Beta diversity heatmap; the smaller coefficient means the top-left sample was more similar with the top-right sample.

Diversity analysis indicated that both α-diversity and β-diversity were higher in the S4 group. The values of Shannon, Simpson, Shannoneven, Simpsoneven, and phylogenetic diversity showed no significant differences (*p* > 0.05) among all groups (Table 5 and Figure 4). The values of ASV, Ace, and Chao1 increased obviously (*p* < 0.05) as the *Schizochytrium* levels raised to 15.00 g/kg diet but decreased with further increasing *Schizochytrium* levels.

**Figure 5:**
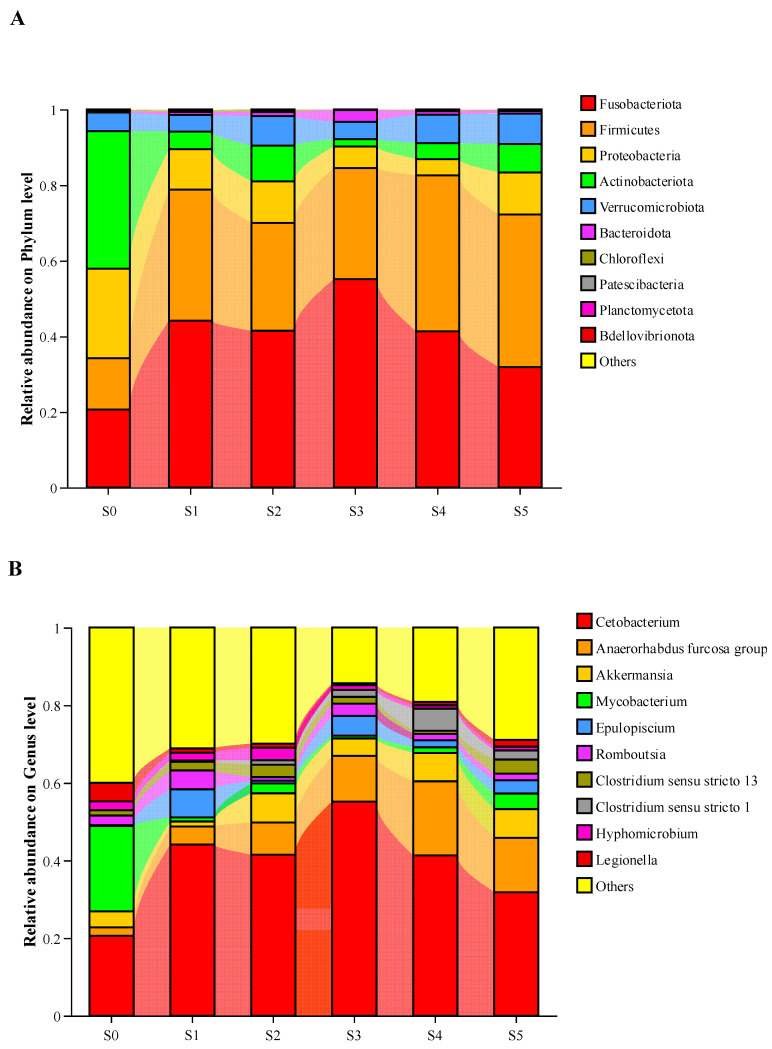

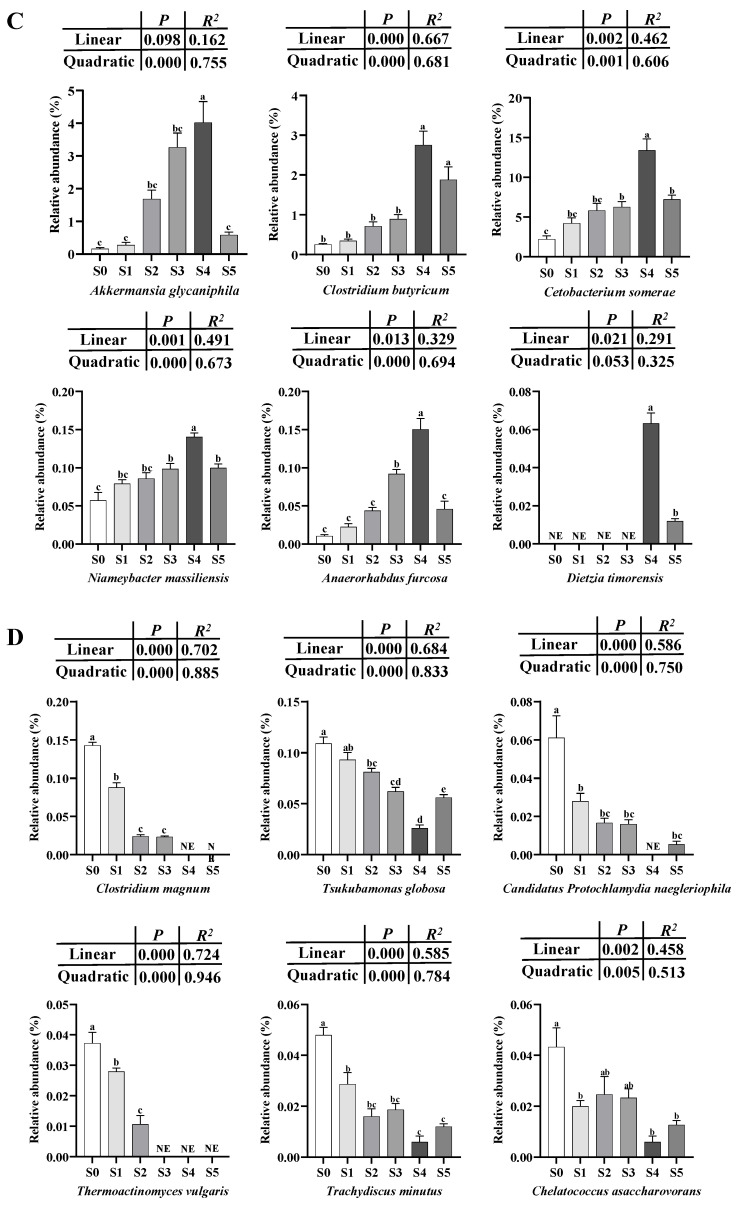
Taxonomy classification of reads at phylum (**A**) and genus (**B**) taxonomic levels of bullfrog tadpoles subjected to different treatments. Only top 10 most abundant (based on relative abundance) bacterial phyla and genera are shown in the figures. Other phyla and genera were all assigned as ‘Others’. (**C**), The upregulated abundance in species; (**D**), The down-regulated abundance in species. Values are mean ± S.E.M of three replications (n = 3). Values with the different superscript letters are significantly different among groups (*p* < 0.05).

Intestinal bacterial composition at the phylum, genus, and species levels is presented in Figure 5. On the phyla level (Figure 5A), Fusobacteriota, Firmicutes, Verrucomicrobiota, and Bacteroidota in tadpoles fed diets with *Schizochytrium* were all more abundant than those of the S0 group, but the opposite trend was found in Proteobacteria, Actinobacteriota, Chloroflexi, Patescibacteria, Planctomycetota, and Bdellovibrionota. The abundances of Firmicutes increased as the *Schizochytrium* levels increased to 15.00 g/kg diet but decreased with further increasing *Schizochytrium* levels. Meanwhile, fish fed the S4 diet have the highest values of Verrucomicrobiota.

On the genus level (Figure 5B), the abundances of *Cetobacterium*, *Anaerorhabdus furcosa*, *Epulopiscium,* and *Clostridium sensu stricto 1* in tadpoles fed diets with *Schizochytrium* were higher than those of the S0 group, but the opposite trend was found in *Legionella*. As for the supplementation of *Schizochytrium*, the abundances of *Anaerorhabdus furcosa* and *Clostridium sensu stricto 1* increased with the increasing levels up to 15.00 g/kg. The lowest values of *Hyphomicrobium* were seen in the S4 group.

On the species level (Figure 5C,D), the abundances of *Clostridium magunm*, *Tsukubamonas globosa*, *Candidatus Protochlamydia naegleriophila*, *Thermooactinomyces vulgaris*, *Trachydiscus minutus*, and *Chelatococcus asaccharovorans* of the S0 group were relatively higher than those of *Schizochytrium* treatments, but the opposite trend was found in *Akkermansia glycanispila*, *Clostridium butyricum*, *Cetobacterium somerae*, *Niameybacter massiliensis*, *Anaerorhabdus furcosa*, and *Dietzia timorensis*. As for the supplementation of *Schizochytrium*, the abundances of *Clostridium magunm* and *Thermooactinomyces vulgaris* declined obviously (*p* < 0.05) with the levels rising up to 20.00 g/kg. The abundances of *Akkermansia glycanispila*, *Clostridium butyricum*, *Cetobacterium somerae*, *Niameybacter massiliensis*, *Anaerorhabdus furcosa*, and *Dietzia timorensis* increased obviously (*p* < 0.05) as the *Schizochytrium* levels raised to 15.00 g/kg diet but decreased with further increasing *Schizochytrium* levels.

### 3.4. The Expression of Genes Related to Growth Performance, Energy Metabolism, and Immune Response

#### 3.4.1. Expression of the Genes Involved in GH-IGF-1 Axes

**Figure 6:**
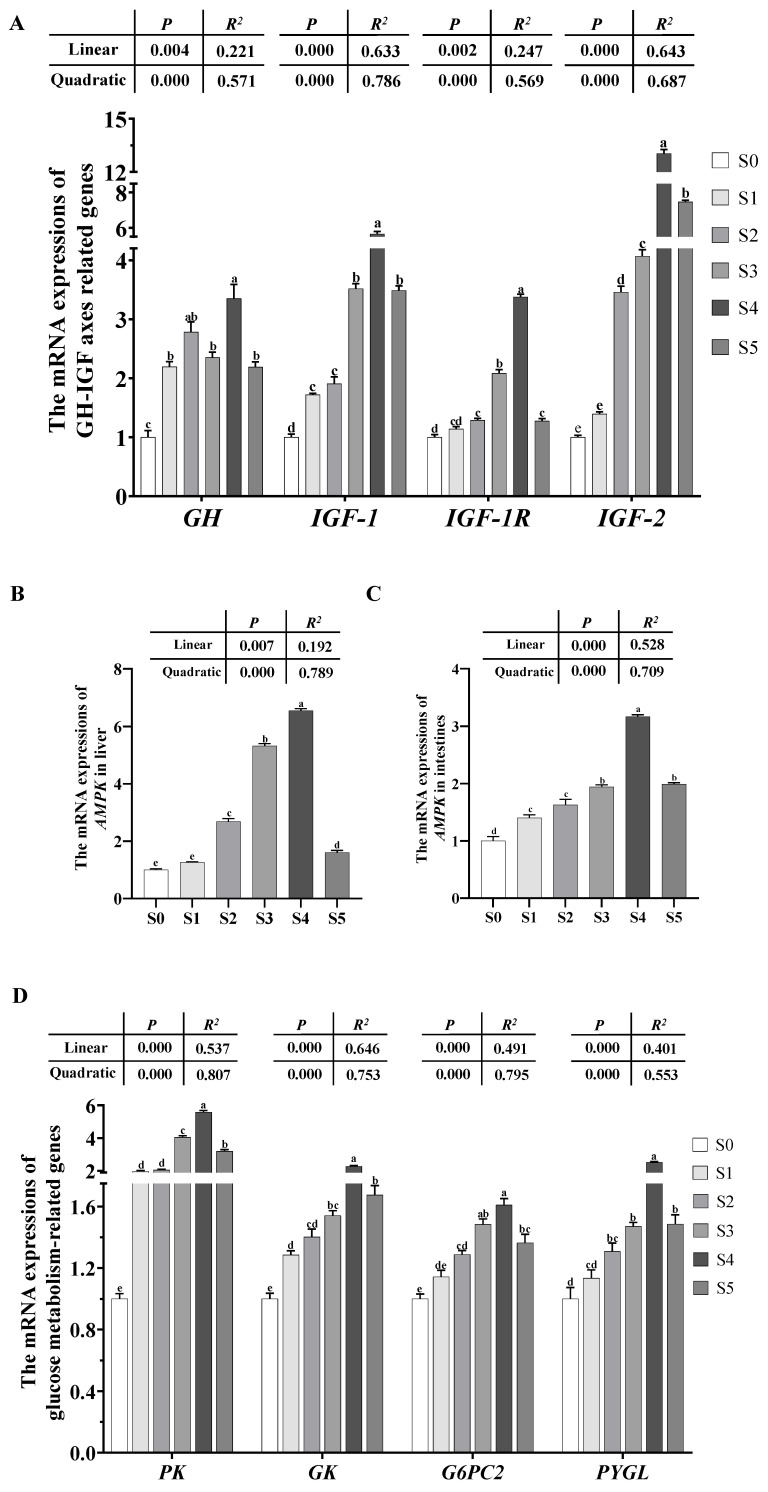

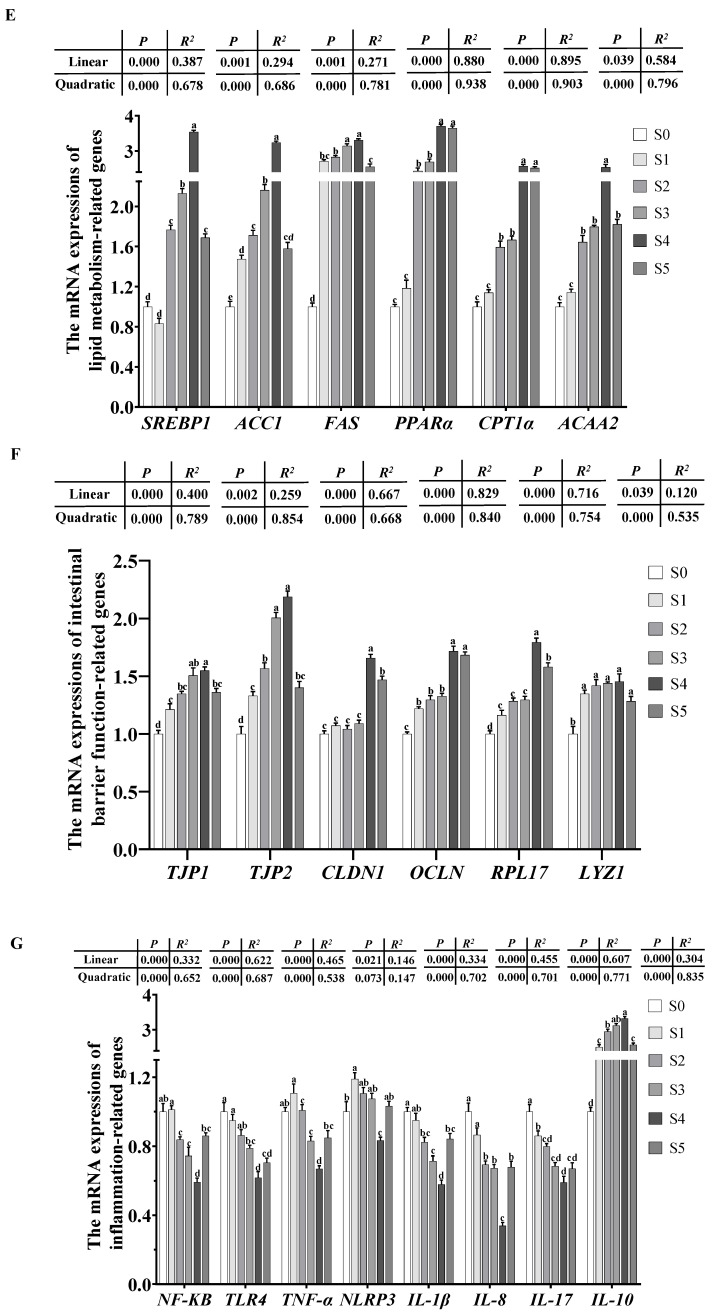
(**A**) Transcriptional levels of *GH-IGF* axes-related genes in brain and liver of bullfrog tadpoles subjected to different treatments. (**B**,**C**) Transcriptional levels of *AMPK* gene in liver and intestine of bullfrog tadpoles subjected to different treatments. (**D**) Transcriptional levels of glucose metabolism-related genes in liver of bullfrog tadpoles subjected to different treatments. (**E**) Transcriptional levels of lipid metabolism-related genes in liver of bullfrog tadpoles subjected to different treatments. (**F**) Transcriptional levels of intestinal barrier function-related genes in intestines of bullfrog tadpoles subjected to different treatments. (**G**) Transcriptional levels of inflammation-related genes in intestines of bullfrog tadpoles subjected to different treatments. *GH*, growth hormone; *IGF-1*, insulin-like growth factor 1; *IGF-1R*, insulin-like growth factor 1 receptor; *IGF-2*, insulin-like growth factor 2; *AMPK*, amp-activated protein kinase; *PK*, pyruvate kinase; *GK*, glycerol kinase; *G6PC2*, glucose-6-phosphatase catalytic, 2; *PYGL*, glycogen phosphorylase, liver; *SREBP1*, sterol-regulatory element binding protein 1; *ACC1*, acetyl-coa carboxylase 1; *FAS*, fatty acid synthase; *PPARα*, peroxisome proliferator-activated receptor alpha; *CPT1α*, carnitine palmitoyl transferase 1α; *ACAA2*, acetyl-coa acyltransferase 2; *TJP1*, tight junction protein 1; *TJP2*, tight junction protein 2; *CLDN1*, claudin 1; *OCLN*, occludin; *RPL17*, ribosomal protein L17; *LYZ1*, lysozyme 1; *NF-KB*, nuclear factor kappa B; *TLR4*, toll-like receptor 4; *TNF-α*, tumor necrosis factor alpha; *NLRP3*, NACHT, LRR, and PYD domains containing protein 3; *IL-1β*, Interleukin 1β; *IL-8*, Interleukin 8; *IL-17*, Interleukin 17; *IL-10*, Interleukin 10. Values are mean ± S.E.M of three replications (n = 3). Values with the different superscript letters are significantly different among groups (*p* < 0.05).

The expression of *GH*, *IGF-1*, *IGF-1R*, and *IGF-2* increased obviously (*p* < 0.05) as *Schizochytrium* levels raised to 15.00 g/kg diet but decreased with further increasing *Schizochytrium* levels (Figure 6A).

#### 3.4.2. Expression of the AMPK Gene

The expression of *AMPK* in the liver and intestine increased obviously (*p* < 0.05) as the *Schizochytrium* levels increased to 15.00 g/kg diet but decreased with further increasing *Schizochytrium* levels (Figure 6B,C).

#### 3.4.3. Expression of the Genes Involved in Glycolipid Metabolism

The expression of *PK*, *GK*, *G6PC2*, *PYGL*, *SREBP1*, *ACC1*, *FAS*, *PPARα*, *CPT1α*, and *ACAA2* in the liver increased obviously (*p* < 0.05) as the *Schizochytrium* levels increased to 15.00 g/kg diet but decreased with further increasing *Schizochytrium* levels (Figure 6D,E).

#### 3.4.4. Expression of the Genes Involved in Intestinal Barrier Function

The expression of *TJP1*, *TJP2*, *CLDN1*, *OCLN*, *RPL17*, and *LYZ1* in the intestine increased obviously (*p* < 0.05) as *Schizochytrium* levels raised to 15.00 g/kg diet but decreased with further increasing *Schizochytrium* levels (Figure 6F).

#### 3.4.5. Expression of the Genes Involved in Inflammation

The expression of *NF-KB*, *TLR4*, *TNF-α*, *NLRP3*, *IL-1β*, *IL-8*, and *IL-17* in the intestine declined obviously (*p* < 0.05) as *Schizochytrium* levels increased to 15.00 g/kg diet but increased with further increasing *Schizochytrium* levels. The expression of IL-10 in intestine rose obviously (*p* < 0.05) as the *Schizochytrium* levels increased to 15.00 g/kg diet but decreased with further increasing *Schizochytrium* levels (Figure 6G).

## 4. Discussion

In the present study, the highest values of final weight, WGR, SGR, FI, PPR, and MR were observed in the S4 group. Additionally, the higher contents of whole-body crude protein and lipids were also observed in S4 group. The results indicated that 15.00 g/kg *Schizochytrium* supplementation could effectively improve the growth performance, metamorphosis, feed utilization, and protein and lipid contents of tadpoles. A key contributing factor appears to be the significantly increased enzyme activities of digestion (e.g., AMS, LPS, PES) and brush border membrane (e.g., Na^+^, K^+^-ATPase, AKP, γ-GT, and CK) in the intestines of the S4 group. These enzymes are crucial for the digestion and absorption of carbohydrates, lipids, and proteins [28,29,30,31].The increased enzyme activities could improve the digestive and absorptive capacity of the intestine, thereby benefitting growth and nutrition utilization and subsequent metamorphosis of tadpoles [32,33]. Moreover, the improvement of intestinal morphology by *Schizochytrium* supplementation should not be neglected. Previous studies displayed that the increase in intestinal microvilli length and mucosal epithelial cell height can improve the digestive and absorptive capacity of the intestine, thereby directly affecting nutrient utilization [22,34,35].

Additionally, intestinal microbial diversity and abundance of bullfrog tadpoles were also significantly modulated by *Schizochytrium* levels. The supplementation of *Schizochytrium* exhibited higher values of ASV, Ace, and Chao1 compared with the S0 group, and the highest levels were observed in the S4 group. The findings indicated that 15.00 g/kg *Schizochytrium* supplementation could increase the intestinal microbial diversity of bullfrogs. Previous studies have shown that the increased microbial diversity is beneficial to the enhancement of immune function in the host [36,37,38]. These findings were further supported by the results of intestinal microbial abundance, where the supplementation of *Schizochytrium* increased the levels of the phyla Fusobacteriota, Firmicutes, Verrucomicrobiotam and Bacteroidota, and the S4 diet-fed tadpoles had the highest abundances of Firmicutes. A previous study has confirmed that Fusobacteriota can improve the integrity of the intestinal barrier, thus enhancing the host’s immune function [39]. Firmicutes and Bacteroidota are both involved in converting complex carbohydrates into beneficial metabolites such as short chain fatty acids (SCFAs), thereby providing energy for host resistance to pathogens [40]. Verrucomicrobiota members can produce antibiotic like substances, which are beneficial for inhibiting the growth and colonization of pathogenic bacteria in the host’s intestine [41]. Meanwhile, Firmicutes was also regarded as a sensitive indicator in the change in host weight, since the increased Firmicutes abundance is often found in hosts who have gained weight [42,43,44]. This result corresponds to the fact that S4-fed bullfrog tadpoles have relatively high growth performance. In addition, the results on the genus and species levels also showed that supplementation of *Schizochytrium* could increase the abundance of beneficial microbiota, such as *Akkermansia glycanispila*, *Clostridium butyricum*, and *Cetobacterium somerae*. Accumulating evidence suggested that all of them can improve intestinal barrier function by degrading mucin to produce SCFAs, thus leading to the enhancement of the host’s immune function [45,46,47,48].

The GH-IGF-1 axis plays an important role in the regulation of growth and feed utilization of aquatic animals. In this study, the highest expression of *GH*, *IGF-1*, *IGF-1R,* and *IGF-2* were observed in the S4 group, which is in line with the results of growth performance. This may be attributed to the fact that DHA-abundant *Schizochytrium* can stimulate the generation of GH, thus leading to an increase in GH expression [49]. Then, the increase in GH induces the release of IGF-1 [50], thereby improving the growth performance of tadpoles.

At the metabolic level, the expression of genes related to energy metabolism (*AMPK*, *PK*, *GK*, *G6PC2*, *PYGL*, *SREBP1*, *ACC1*, *FAS*, *PPARα*, *CPT1α*, and *ACAA2*) in the liver upregulated obviously as the *Schizochytrium* levels increased to 15.00 g/kg diet. The results indicated that the S4 diets significantly enhance glycolysis, gluconeogenesis, glycogenolysis, lipogenesis, and lipolysis of tadpoles. According to a previous study, AMP-activated protein kinase (AMPK) is a highly conserved sensor of cellular energy status that can regulate the energy metabolism of organisms [51], and DHA-rich food can activate the AMPK [52]. The activation of *AMPK* can participate in a series of physiological consequences concerning glycolipid metabolism, such as (1) the enhancement of glycolysis and glycogenolysis by upregulating *GK* and *PYGL* activities, respectively [53,54]; and (2) the increase in lipolysis by activating *PPARα* and *PPARα*-downstream target genes (*CPT1α* and *ACAA2*) [55]. In addition, DHA has been demonstrated to upregulate expression of lipogenesis-related genes, thereby resulting in an increase in lipid contents [56]. For gluconeogenesis, this may be attributed to the enhanced glycolysis that could result in an increase in pyruvate content, which in turn stimulates gluconeogenesis characterized by the upregulation of *G6PC2* expression [57].

Intestinal health partly depends on intestinal barrier function which is associated with the activities of tight junction proteins, such as claudins and occlusions [58]. In this study, the mRNA levels of *AMPK* and tight junction proteins (e.g., *TJP1*, *TJP2*, *CLDN1*, *OCLN*, *RPL17*, and *LYZ1*) in the intestine increased significantly as the *Schizochytrium* levels increased to 15.00 g/kg diet, indicating that the S4 diet significantly enhanced the intestinal barrier function of tadpoles. The results may be due to the fact that the activation of AMPK could promote tight junction-related protein synthesis, thereby leading to the enhancement of intestinal barrier function characterized by the upregulation of tight junction proteins expression [59,60]. According to previous research, the enhanced intestinal barrier function can enhance the immunity of aquatic animals [61]. Consistent with an enhanced barrier and AMPK’s role in modulating redox and inflammatory states, the S4 group exhibited significantly elevated activities of antioxidant enzymes (SOD, CAT) and a marked down-regulation of pro-inflammatory genes (*NF-KB*, *TLR4*, *TNF-α*, *NLRP3*, *IL-1β*, *IL-8*, *IL-17*). AMPK can activate the Nrf2-mediated antioxidant pathway and inhibit NF-kB signaling by deacetylating the p65 subunit [62,63,64], thereby collectively reducing intestinal inflammation and oxidative stress.

## 5. Conclusions

In conclusion, dietary supplementation of *Schizochytrium* significantly enhanced bullfrog tadpole growth and metamorphosis by simultaneously improving digestive function, intestinal barrier integrity, and intermediate metabolism, while attenuating intestinal inflammation. Optimal *Schizochytrium* levels for bullfrog tadpoles were estimated to be 13.27 g/kg diet. This study provided a practical nutritional strategy for sustainable bullfrog aquaculture. A limitation of this work was that the precise causal relationships between the reshaped gut microbiota and the observed metabolic benefits warrant further investigation. Future studies should focus on validating these findings under commercial farming conditions and elucidating the underlying molecular mechanisms in greater detail.

## Figures and Tables

**Table 1:** Formulation and proximate composition of the different experimental diets (% dry matter basis).

	Groups					
Items	S0	S1	S2	S3	S4	S5
*Formulation* (%)						
Fish meal	10.0	10.0	10.0	10.0	10.0	10.0
Chicken meal	8.0	8.0	8.0	8.0	8.0	8.0
Dephenolized cottonseed protein	15.0	15.0	15.0	15.0	15.0	15.0
Rapeseed meal	16.0	16.0	16.0	16.0	16.0	16.0
Soybean meal	30.0	30.0	30.0	30.0	30.0	30.0
Corn starch	14.5	14.5	14.5	14.5	14.5	14.5
*Schizochytrium* (g/kg)	-	2.0	5.0	10.0	15.0	20.0
Fish oil	1.5	1.5	1.5	1.5	1.5	1.5
Soybean oil	1.5	1.5	1.5	1.5	1.5	1.5
Calcium biphosphate	1.0	1.0	1.0	1.0	1.0	1.0
Choline chloride	0.5	0.5	0.5	0.5	0.5	0.5
Premix *	1.0	1.0	1.0	1.0	1.0	1.0
DL-lysine	0.5	0.5	0.5	0.5	0.5	0.5
DL-methionine	0.5	0.5	0.5	0.5	0.5	0.5
*Proximate analysis* (% dry matter basis)						
Moisture	8.14	7.87	8.03	7.93	8.01	7.90
Crude protein	29.17	28.14	28.9	29.16	28.77	29.13
Crude lipid	4.23	4.53	4.51	4.43	4.49	4.52
Ash	15.53	15.01	14.95	14.90	15.17	15.01

* Notes: vitamin premix (mg/kg) [vitamin A 500,000 IU, vitamin D3 50,000 IU, vitamin E2 500 mg, vitamin K3 1000 mg, vitamin B1 5000 mg, vitamin B2 5000 mg, vitamin B6 5000 mg. vitamin B12 5000 ug, inositol 25,000 mg, pantothenic acid 10,000 mg, choline 100,000 mg. Niacin 25,000 mg, folic acid 1000 mg, biotin 250 mg, vitamin C 10,000 mg]; mixed minerals (g/kg) contains CaCO_3_ 314.0 g, KH_2_PO_4_ 469.3 g, MgSO_4_·7H_2_O 147.4 g, NaC1 49.8 g, Fe(II)gluconate 10.9 g, MnSO_4_·7H_2_O 3.12 g, ZnSO_4_-7H_2_O 4.67 g, CuSO_4_-5H_2_O 0.62 g, KI 0.16 g, CoCl_2_-6H_2_O 0.08 g, NH_4_ molybdate 0.06 g, NaSeO_3_ 0.02 g.

**Table 2:** Primer sequences for qPCR.

Target Genes	Forward (5′-3)	Reverse (5′-3)	Accession Numbers or References
*GH*	TAGACCTCCTCCGCTTCTCC	ACCGTAGTTCCGGACGTTTC	AY251538.1
*AMPK*	GGAGGTGCTCAGCTGCTTGT	AATGAATCGGGCGGGCTTGT	PIO34006.1
*IGF-* *1*	CAGTTTGTATGTGGAGACAGAGGCT	CAGTACATCTCTAGCCTCCTCAGA	XM040344286.1
*IGF-* *1* *R*	TCCGTCTGTTAGGCGTTGT	AGGGTTGTTCTCGGCATCT	KF819507
*IGF-* *2*	TGCCACTGCATTGCCATCTCT	TCTATTTGCCTCACCCACCGC	XM040328472.1
*PK*	TCCTTCATTCGCAAGGCAGC	TGATATTCTTTCCTTTCTCCCCGA	XM040343042.1
*G6PC2*	CTGCTCCAGTCATTGAACAGTTTC	CCCCATAGCGTGACCTGAAG	LH203031
*PYGL*	CTCGAGTGCTCTATCCCAATGAT	ACCACAAAGTACTCCTGCTTCAGA	LH181312
*GK*	AACGCTTTGAGCCACAGATTAAT	CTGCTTTTTTCCATCGAGCAT	LH193866
*SREBP1*	CCACCGCCTGCACCAA	CACTCAGCGCCATGTTGATG	LH362962
*ACC1*	GTTAAAGCTGCCATCCTCACTGT	TGTCCGTCTGGCTAAGATGGT	LH212450
*FAS*	CCTCCACGCCAGAACAAGAT	GATATTTTTATGAGTGGACATTGTATCGA	LH228595
*PPARα*	CCCGACATTCGATGTTTAGAGATT	CCAGCCCATCTTCTATCACCTT	LH193621
*CPT1α*	TGATTGGCAAAATCAAAGAACATC	AATGCTCTGACCCTGGTGAGA	LH022414
*ACAA2*	TGTTAGCAGGAGGTAAAAGGAAAGTC	ATGCGTTCAACAGTTTTTGGAA	LH119205
*LYZ1*	ATGGAAAGACAGCAGGAG	TCCAGAAGTATACGATCC	[25]
*TJP1*	TACGAGAAAGTGGTGCTC	CCTCACTCACAAGACTTG	[25]
*TJP2*	GACCGTAGAAGTGCATAC	CCTGATCTCTTCCATAGC	[25]
*CLDN1*	TTGTCCCTCCTGTCTGTCATCT	ACTGGAGAGGTGAAGTAGTGG	XM040320982.1
*OCLN*	TGCAACGATCGTGTACAC	GTGAATAGAACTGGTTGC	[25]
*RPL17*	CAAACAGTGGGACTGGACACA	TTCTTCAGCATGTGCAGGAGGAAT	XM040336449.1
*NF-KB*	AGGAACAAGATCATCATAGAGGAC	TCAGAAAGAGGGGCCAAGTG	XM040329914.1
*TLR4*	ACACCTTCAAGGCTCGCTAC	ATGATGGGTCGCCATCTTCC	XM040322748.1
*NLRP3*	ATCACCACAAGGTCACTGGC	TGATTGGCCAGAGCACACAGC	XM040352091.1
*TNF-α*	ACAGACTTGATGGACCTC	AGTTCCAGCTTCGATGTG	[25]
*IL-1β*	TCATTCGGGACAGCAGGCAGAA	GCTTCACTGGCACGGTTGTTCT	[14]
*IL-8*	ACCCTTACCCTCTTCCTGCT	TCAGCTTCACACACTGGCA	XM040335899.1
*IL-10*	GGAAGTTGTCAGCGGGCTAT	GCCCTCTTGTGCATGTGGTA	XM040339663.1
*IL-17*	TGATAGTCACGCACTGAGTCCG	ATGTTCACCAGCCAGTCAATGC	[14]
*β-actin*	CATCCTTCTTGGGTATGGAATCA	TGGCATACAGGTCCTTACGGATA	AB094353

*GH*, growth hormone; *AMPK*, amp-activated protein kinase; *IGF-1*, insulin-like growth factor 1; *IGF-1R*, insulin-like growth factor 1 receptor; *IGF-2*, insulin-like growth factor 2; *PK*, pyruvate kinase; *G6PC2*, glucose-6-phosphatase catalytic, 2; *PYGL*, glycogen phosphorylase, liver; *GK*, glycerol kinase; *SREBP1*, sterol-regulatory element binding protein 1; *ACC1*, acetyl-coa carboxylase 1; *FAS*, fatty acid synthase; *PPARα*, peroxisome proliferator-activated receptor alpha; *CPT1α*, carnitine palmitoyl transferase 1α; *ACAA2*, acetyl-coa acyltransferase 2; *LYZ1*, lysozyme 1; *TJP1*, tight junction protein 1; *TJP2*, tight junction protein 2; *CLDN1*, claudin 1; *OCLN*, occluding; *RPL17*, ribosomal protein L17; *NF-KB*, nuclear factor kappa B; *TLR4*, toll-like receptor 4; *NLRP3*, NACHT, LRR, and PYD domains containing protein 3; *TNF-α*, tumor necrosis factor alpha; *IL-1β*, Interleukin 1β; *IL-8*, Interleukin 8; *IL-10*, Interleukin 10; *IL-17*, Interleukin 17.

**Table 3:** Growth performance and proximate composition of bullfrog tadpoles subjected to different treatments.

	Groups						Polynomial Contrasts (*p*, R^2^)	
Items	S0	S1	S2	S3	S4	S5	Linear	Quadratic
Initial weight (g)	0.04 ± 0.00	0.04 ± 0.00	0.04 ± 0.00	0.04 ± 0.00	0.04 ± 0.00	0.04 ± 0.00	0.524, 0.026	0.707, 0.045
Final weight (g)	4.69 ± 0.02 ^e^	5.34 ± 0.03 ^d^	5.41 ± 0.05 ^c,d^	5.66 ± 0.06 ^b^	6.15 ± 0.06 ^a^	5.58 ± 0.05 ^b,c^	0.001, 0.522	0.000, 0.826
WGR † (%)	13,066.15 ± 148.16 ^c^	15,028.36 ± 197.53 ^b^	15,127.35 ± 85.10 ^b^	15,469.35 ± 64.80 ^b^	16,173.45 ± 202.30 ^a^	15,405.86 ± 83.80 ^b^	0.002, 0.474	0.000, 0.775
SGR § (%/day)	5.42 ± 0.01 ^c^	5.58 ± 0.01 ^b^	5.58 ± 0.01 ^b^	5.61 ± 0.00 ^a,b^	5.66 ± 0.01 ^a^	5.60 ± 0.01 ^b^	0.002, 0.467	0.000, 0.768
FCR ¶	2.55 ± 0.04 ^a^	2.51 ± 0.05 ^a^	2.47 ± 0.04 ^a,b^	2.44 ± 0.03 ^a,b^	2.24 ± 0.05 ^c^	2.31 ± 0.02 ^b,c^	0.000, 0.654	0.000, 0.670
FI (g/bullfrog tadpoles/d) #	0.13 ± 0.00 ^c^	0.15 ± 0.00 ^a,b^	0.15 ± 0.00 ^a,b^	0.15 ± 0.00 ^a^	0.15 ± 0.00 ^a^	0.14 ± 0.00 ^b^	0.151, 0.124	0.000, 0.697
CF ∫	0.96 ± 0.02	0.97 ± 0.03	0.92 ± 0.03	0.98 ± 0.02	0.98 ± 0.04	0.91 ± 0.03	0.725, 0.008	0.195, 0.196
PPR **‽** (%)	79.17 ± 2.20 ^b^	80.83 ± 3.00 ^a,b^	82.50 ± 3.82 ^a,b^	86.67 ± 3.33 ^a,b^	93.33 ± 1.67 ^a^	85.00 ± 1.44 ^a,b^	0.017, 0.307	0.011, 0.452
MR1 ↑ (%)	8.33 ± 2.20 ^b^	10.00 ± 1.44 ^b^	13.33 ± 0.83 ^b^	17.50 ± 2.89 ^a,b^	23.33 ± 1.67 ^a^	17.50 ± 2.50 ^a,b^	0.001, 0.533	0.000, 0.679
*Proximate analysis* (% dry matter basis)								
Moisture	80.16 ± 2.64	76.69 ± 2.90	77.02 ± 1.64	75.23 ± 2.49	76.62 ± 1.53	79.15 ± 1.88	0.882, 0.001	0.224, 0.181
Crude protein	76.53 ± 0.91 ^c^	81.84 ± 0.21 ^a,b^	80.68 ± 1.03 ^b,c^	84.02 ± 0.08 ^a,b^	84.56 ± 1.46 ^a,b^	85.19 ± 0.78 ^a^	0.000, 0.627	0.000, 0.712
Crude lipid	14.56 ± 0.14 ^a,b^	14.4 ± 0.22 ^b^	14.51 ± 0.21 ^b^	15.91 ± 0.33 ^a^	15.03 ± 0.15 ^a,b^	15.5 ± 0.52 ^a,b^	0.015, 0.317	0.034, 0.362
Ash	12.37 ± 0.51	12.08 ± 0.85	12.98 ± 0.39	11.9 ± 1.54	12.3 ± 1.34	12.93 ± 1.63	0.783, 0.005	0.907, 0.013

Values are mean ± S.E.M of three replications (n = 3). Means in the same line with different superscripts are significantly different (*p* < 0.05). † Weight gain rate (WGR, %) = (Wt − W0) × 100/W0. §: Specific growth rate (SGR, %/day) = (LnWt − LnW0) × 100/T, where W0 and Wt are the initial and final body weights, and T is the culture period in days. ¶: Feed conversion ratio (FCR) = feed consumption (g)/bullfrog tadpole weight gain (g). #: Feed intake (FI) (g/bullfrog tadpoles/d) = total feed intake (g)/[total number of bullfrog tadpoles× days reared]. ∫: Condition factor (CF) = body weight(g) × 100/total body length (cm)^3^. **‽**: Post-premetamorphosis rate (PPR) = number of bullfrog tadpoles with hind limb buds at 90 days/number of bullfrog tadpoles at the start of the experiment. ↑: Metamorphosis rate (MR) = number of metamorphosed bullfrog tadpoles/initial number of tadpoles × 100.

**Table 4:** Intestinal enzymes activities and antioxidant parameters of bullfrog tadpoles subjected to different treatments.

	Groups						Polynomial Contrasts (*p*, R^2^)	
Items	S0	S1	S2	S3	S4	S5	Linear	Quadratic
Amylase (U/mg protein)	0.24 ± 0.01 ^d^	0.27 ± 0.00 ^c,d^	0.28 ± 0.01 ^c^	0.29 ± 0.01 ^c^	0.38 ± 0.01 ^a^	0.33 ± 0.01 ^b^	0.000, 0.563	0.000, 0.601
Lipase (U/g protein)	8.20 ± 0.30 ^e^	11.17 ± 0.39 ^d^	11.65 ± 0.12 ^d^	13.38 ± 0.14 ^c^	19.6 ± 0.25 ^a^	15.8 ± 0.34 ^b^	0.000, 0.852	0.000, 0.806
Protease (U/mg protein)	1.03 ± 0.03 ^e^	1.36 ± 0.04 ^d^	1.72 ± 0.03 ^c^	1.99 ± 0.03 ^b^	2.46 ± 0.03 ^a^	2.01 ± 0.02 ^b^	0.000, 0.686	0.000, 0.919
Na^+^, K^+^-ATPase (U/g protein)	44.89 ± 1.81 ^d^	59.55 ± 2.78 ^cd^	78.19 ± 2.77 ^c^	121.00 ± 5.03 ^b^	159.59 ± 12.80 ^a^	143.56 ± 3.67 ^a,b^	0.000, 0.767	0.000, 0.827
AKP (U/g protein)	55.28 ± 0.18 ^c^	54.10 ± 0.78 ^b,c^	60.34 ± 0.75 ^b,c^	63.28 ± 0.91 ^a,b^	68.83 ± 0.57 ^a^	60.34 ± 0.85 ^b,c^	0.002, 0.446	0.000, 0.766
γ-GT (U/g protein)	79.18 ± 4.85 ^c^	76.79 ± 1.66 ^c^	99.78 ± 3.53 ^b^	122.77 ± 4.97 ^a^	140.00 ± 5.37 ^a^	73.09 ± 3.04 ^c^	0.251, 0.081	0.000, 0.709
CK (U/mg protein)	1.47 ± 0.08 ^c^	1.85 ± 0.02 ^b^	1.95 ± 0.03 ^a,b^	1.99 ± 0.02 ^a,b^	2.12 ± 0.03 ^a^	2.06 ± 0.04 ^a^	0.000, 0.476	0.000, 0.638
SOD (U/mg protein)	555.75 ± 5.33 ^c^	576.85 ± 17.78 ^b,c^	600.13 ± 15.64 ^b,c^	629.39 ± 9.12 ^a,b^	669.74 ± 11.9 ^a^	580.32 ± 7.12 ^b,c^	0.064, 0.202	0.000, 0.666
CAT (U/mg protein)	10.35 ± 0.17 ^d^	11.32 ± 0.35 ^c^	11.89 ± 0.16 ^c^	12.92 ± 0.16 ^b^	14.01 ± 0.07 ^a^	11.35 ± 0.07 ^c^	0.000, 0.215	0.000, 0.703
T-AOC (mmol/g protein)	0.03 ± 0.00	0.03 ± 0.00	0.03 ± 0.00	0.03 ± 0.00	0.03 ± 0.00	0.03 ± 0.00	0.703, 0.009	0.762, 0.036
MDA (nmol/mg protein)	4.59 ± 0.05 ^a^	4.39 ± 0.08 ^a,b^	4.28 ± 0.04 ^b,c^	4.17 ± 0.07 ^b,c^	3.68 ± 0.05 ^d^	4.10 ± 0.06 ^c^	0.000, 0.461	0.000, 0.765

Na^+^, K^+^-ATPase, sodium–potassium adenosine triphosphatase; AKP, alkaline phosphatase; γ-GT, γ-glutamyl transferase; CK, creatine kinase; SOD, superoxide dismutase; CAT, catalase; T-AOC, total antioxidant capacity; MDA, malondialdehyde. Values are mean ± S.E.M of three replications (n = 3). Means in the same line with different superscripts are significantly different (*p* < 0.05).

**Table 5:** The alpha diversity indices of intestinal microbiota of bullfrog tadpoles subjected to different treatments.

	Groups						Polynomial Contrasts (*p*, R^2^)	
Items	S0	S1	S2	S3	S4	S5	Linear	Quadratic
ASV	248.67 ± 3.84 ^a,b^	271.33 ± 38.92 ^a,b^	182.00 ± 25.32 ^b^	216.67 ± 19.97 ^a,b^	352.00 ± 31.34 ^a^	234.00 ± 41.14 ^a,b^	0.457, 0.035	0.761, 0.036
Ace	250.33 ± 3.18 ^a,b^	274.33 ± 39.96 ^a,b^	182.33 ± 25.12 ^b^	216.67 ± 19.97 ^a,b^	353.67 ± 31.86 ^a^	235.00 ± 41.04 ^a,b^	0.474, 0.033	0.774, 0.034
Chao1	250.33 ± 3.18 ^a,b^	274.33 ± 39.96 ^a,b^	182.33 ± 25.12 ^b^	216.67 ± 19.97 ^a,b^	353.67 ± 31.86 ^a^	235.00 ± 41.04 ^a,b^	0.474, 0.033	0.774, 0.034
Shannon	2.98 ± 0.12	2.34 ± 0.31	2.21 ± 0.34	2.46 ± 0.18	3.31 ± 0.13	2.62 ± 0.55	0.483, 0.031	0.699, 0.047
Simpson	0.14 ± 0.04	0.28 ± 0.07	0.28 ± 0.07	0.18 ± 0.01	0.10 ± 0.02	0.22 ± 0.13	0.600, 0.018	0.874, 0.018
Shannoneven	0.54 ± 0.02	0.42 ± 0.04	0.42 ± 0.05 ^b^	0.46 ± 0.03	0.56 ± 0.02 ^a^	0.48 ± 0.09	0.541, 0.024	0.730, 0.041
Simpsoneven	0.03 ± 0.01	0.01 ± 0.00	0.02 ± 0.00	0.03 ± 0.00	0.03 ± 0.01	0.03 ± 0.01	0.387, 0.047	0.527, 0.082
Phylogenetic diversity	28.64 ± 0.11	31.04 ± 5.40	21.61 ± 2.32	25.54 ± 1.52	35.86 ± 3.03	26.28 ± 3.72	0.719, 0.008	0.918, 0.011

ASV, Amplicon sequence variant. Values are mean ± S.E.M of three replications (n = 3). Means in the same line with different superscripts are significantly different (*p* < 0.05).

## Data Availability

Data is contained within the article.

## Abbreviations

The following abbreviations are used in this manuscript:

*GH*	Growth hormone
*AMPK*	Amp-activated protein kinase
*IGF-1*	Insulin-like growth factor 1
*IGF-1R*	Insulin-like growth factor 1 receptor
*IGF-2*	Insulin-like growth factor 2
*PK*	Pyruvate kinase
*G6PC2*	Glucose-6-phosphatase catalytic, 2
*PYGL*	Glycogen phosphorylase, liver
*GK*	Glycerol kinase
*SREBP1*	Sterol-regulatory element binding protein 1
*ACC1*	Acetyl-coa carboxylase 1
*FAS*	Fatty acid synthase
*PPARα*	Peroxisome proliferator-activated receptor alpha
*CPT1α*	Carnitine palmitoyl transferase 1α
*ACAA2*	Acetyl-coa acyltransferase 2
*LYZ1*	Lysozyme 1
*TJP1*	Tight junction protein 1
*TJP2*	Tight junction protein 2
*CLDN1*	Claudin 1
*OCLN*	Occluding
*RPL17*	Ribosomal protein L17
*NF-KB*	Nuclear factor kappa B
*TLR4*	Toll-like receptor 4
*NLRP3*	NACHT, LRR, and PYD domains-containing protein 3
*TNF-α*	Tumor necrosis factor alpha
*IL-1β*	Interleukin 1β
*IL-8*	Interleukin 8
*IL-10*	Interleukin 10
*IL-17*	Interleukin 17
WGR	Weight gain rate
SGR	Specific growth rate
FCR	Feed conversion ratio
FI	Feed intake
CF	Condition factor
PPR	Post-premetamorphosis rate
MR	Metamorphosis rate
Na^+^, K^+^-ATPase	Sodium-potassium adenosine triphosphatase
AKP	Alkaline phosphatase
γ-GT	γ-glutamyl transferase
CK	Creatine kinase
SOD	Superoxide dismutase
CAT	Catalase
T-AOC	Total antioxidant capacity
MDA	Malondialdehyde
ASV	Amplicon sequence variant
CT	Connective tissue
ME	Mucosal epithelial cells
M	Microvilli
N	Cell nucleus
PCoA	Principal co-ordinates analysis
NMDS	Non-metric multidimensional scaling
